# Supplementary material for: Extended anharmonic collapse of phonon dispersions in SnS and SnSe
Source: Nat Commun. 2020 Sep 4;11:4430. doi: 10.1038/s41467-020-18121-4 (PMC7474096; doi:10.1038/s41467-020-18121-4)
Supplement: Supplementary file 1 — Supplementary Information [file 41467_2020_18121_MOESM1_ESM.pdf]

Supplementary Information - Extended anharmonic collapse of phonon dispersions in SnS and SnSe

T. Lanigan-Atkins,[1,*] S. Yang,[1,*] J. L. Niedziela,[2] D. Bansal,[1] A. F. May,[2] A. A. Puretzky,[3] J. Y. Y. Lin,[4] D. M. Pajerowski,[4] T. Hong,[4] S. Chi,[4] G. Ehlers,[4] and O. Delaire[1,5,6,†]

[1] *Department of Mechanical Engineering and Materials Science, Duke University, Durham, NC, USA*
[2] *Materials Science and Technology Division, Oak Ridge National Laboratory, Oak Ridge, TN, USA*
[3] *Center for Nanophase Materials Sciences, Oak Ridge National Laboratory, Oak Ridge, TN 37831, USA*
[4] *Neutron Scattering Division, Oak Ridge National Laboratory, Oak Ridge, TN, USA*
[5] *Department of Physics, Duke University, Durham, NC, USA*
[6] *Department of Chemistry, Duke University, Durham, NC, USA*
(Dated: August 5, 2020)

1. SUPPLEMENTARY NOTES

Supplementary Note 1: Thermoelectric materials

Thermoelectric materials enable the direct conversion of heat into electrical energy. Their performance is characterized by the thermoelectric figure-of-merit zT:

$$zT = \frac{\sigma S^2 T}{\kappa_{\rm el} + \kappa_{\rm lat}}, \tag{1}$$

where S is the Seebeck coefficient, σ is the electrical conductivity and $\kappa_{\rm el}/\kappa_{\rm lat}$ is the electronic/lattice component of thermal conductivity. In semiconductor materials typically $\kappa_{\rm lat} >> \kappa_{\rm el}$.

Supplementary Note 2: Symmetry changes across the Pnma-Cmcm phase transition

The high-temperature orthorhombic Cmcm phase is a layered structure composed of rocksalt slabs. The low-temperature Pnma phase below $T_{\rm C}$, also orthorhombic, results from a distortion of the high-temperature parent phase corresponding mainly to displacements (off-centering) of Sn atoms along the c axis, breaking a mirror plane symmetry perpendicular to this axis [1, 2]. The magnitude of the Sn off-centering constitutes an order parameter for the transition, which gradually increases over several hundred degrees below $T_{\rm C}$. Cooling from the Cmcm phase to the Pnma phase results in a doubling of the unit cell size, and a new set of superlattice Bragg peaks at wave-vectors corresponding to the Y zone-boundary points of the parent Cmcm phase. We note that while Pnma is the notation most commonly used to describe the low-T phase, it may equivalently be described as Pbnm (both space group 62), which may facilitate the comparison with the Cmcm phase [2]. Pbnm is a subgroup of Cmcm of index 2 (preserving half of its symmetry elements), and this enables a second-order transition between the two phases. For detailed studies of the structural evolution of SnSe and SnS see Refs. [1–4].

Supplementary Note 3: Inelastic neutron scattering

The dynamical structure factor S$(\mathbf{Q}, \omega)$ was obtained as:

$$S(\mathbf{Q},\omega) = \Sigma_s \Sigma_\tau \frac{1}{\omega_s} \left| \Sigma_d \frac{\overline{b_d}}{\sqrt{M_d}} \exp(-\mathrm{W_d}) \exp(\mathrm{i}\mathbf{Q}\cdot \mathrm{r_d})(\mathbf{Q}\cdot \mathbf{e}_{\rm ds}) \right|^2 \times \langle n_s + 1 \rangle \delta(\omega - \omega_s)\delta(\mathbf{Q} - \mathbf{q} - \tau), \tag{2}$$

*These authors contributed equally to this work
†Electronic address: olivier.delaire@duke.edu

where s is branch index, τ is reciprocal lattice vector, ω is phonon frequency, b_d is neutron scattering length, M_d is atomic mass, W_d is the Debye-Waller factor, $\mathbf{Q} = \mathbf{k}_\mathrm{i} - \mathbf{k}_\mathrm{f}$ with $\mathbf{k}_\mathrm{i}/\mathbf{k}_\mathrm{f}$ being incident/final neutron wavevector, r_d is the atomic coordinate and $\mathbf{e}_{ds}$ is the phonon eigenvector.

The Bose-Einstein occupation factor n is defined as

$$n = \frac{1}{e^{\frac{\hbar\omega}{k_B T}} - 1}, \tag{3}$$

where k_B is Boltzmann's constant and $\hbar$ is the reduced Planck's constant. The dynamical susceptibility is obtained from:

$$\chi''(\mathbf{Q}, E) = \frac{S(\mathbf{Q}, E)}{(n + \frac{1}{2} \pm \frac{1}{2})}, \tag{4}$$

where the positive/negative sign is used for phonon creation/annihilation. By considering χ'' instead of $S(\mathbf{Q}, E)$ we correct for differences in phonon occupancies with temperature. This allows us to focus on the intrinsic behavior with regard to phonon intensities, such as changes in the phonon polarization vectors.

In fits of INS spectra to extract phonon energies, the phonon profiles were modeled as damped harmonic oscillators (DHO) whilst Bragg peaks and the signal from incoherent elastic scattering were treated as Gaussians. The DHO is defined as

$$I_{\mathrm{DHO}}(E) \propto \frac{\Gamma_{\mathrm{LW}} E}{(E^2 - E_0^2)^2 + (\Gamma_{\mathrm{LW}} E)^2}, \tag{5}$$

where Γ_{LW} is the damping parameter which can also be referred to as the linewidth and E_0 is the bare (undamped) phonon frequency.

Supplementary Note 4: Raman

Group theory can be utilized to identify which modes are Raman- (R) or Infra-red- (IR) active depending on whether they change the polarizability (Raman-active) or the dipole moment (IR-active) [5]. For the low temperature phase which crystallizes in the Pnma structure the following activities are predicted:

$$\Gamma = 4B_{1\mathrm{u}}(IR) + 2B_{2\mathrm{u}}(IR) + 4B_{3\mathrm{u}}(IR) + 4A_\mathrm{g}(R) + 2B_{1\mathrm{g}}(R) + 4B_{2\mathrm{g}}(R) + 2B_{3\mathrm{g}}(R). \tag{6}$$

We measured single crystals that cleaved in the $b-c$ plane with the incident laser perpendicular to this plane. Due to the existence of temperature gradients within the Linkam stage a method of temperature calibration was devised. During measurements samples were mounted on a sapphire disk which could be used for calibration purposes. Previous work has established the temperature dependence of phonon modes of sapphire to a high degree of accuracy [6]. At each temperature where the sample was measured a separate spectrum was collected for sapphire in order to calibrate the temperature.

Supplementary Note 5: Force-constants analysis

Supplementary Table 2 lists the force constants (Sn-S bonds shown in Supplementary Figure 27-a) critical to the Pnma-Cmcm transition in SnS. The strongest bond is d_1 which points perpendicular to the b-c plane. Bonds d_2 and d_3 are the in-plane (b-c plane) bonds with d_2 describing the shorter bond and d_3 corresponding to the longer bond. The d_2 and d_3 bonds are equivalent in the Cmcm phase but as the symmetry is lowered upon cooling through T_C they become different. From 0 K to 600 K the $\Phi_{11}^{(2)}$ component of d_1, which is out-of-plane, and the $\Phi_{22}^{(2)}$ and $\Phi_{33}^{(2)}$ elements of d_2, which in-plane, become much weaker. This reflects that d_1 is changing mostly along a whereas for d_2 the changes are in the *b*-*c* plane. The d_3 force constants are notably lower than those for d_1 and d_2 and the changes are also small. The changes in force-constants for different bonds are consistent with the reported evolution of bond lengths with temperature [7]. The softening of the TO_c phonon mode mainly arises from the softening of in-plane force-constants, especially associated with the d_2 bond. The in-plane bonds (d_2 and d_3) change significantly across the phase transition (600-800 K) whereas the out-of-plane d_1 bond shows little change. Thus, the in-plane d_2 and d_3 bonds affect the phonon softening close to transition temperatures, while d_1 affects the phonon softening from 0 K to 600 K in simulations.

Supplementary Note 6: Phonon eigenvectors

The eigenvectors obtained from first-principles simulations for the soft mode in the Pnma and Cmcm phases are shown in Supplementary Figure 27. The soft mode eigenvector accounts for most of the Pnma-Cmcm distortion but additional modes also contribute, similarly to the case of SnSe [8]. The structural distortion from Pnma to Cmcm involves Sn and S atoms moving mainly along the c direction [1], with a more limited component along a. This displacement pattern matches well with the eigenvector of the TO_c mode in the Pnma phase. For this mode, the main eigenvector component corresponds to Sn motions. The eigenvector of the zone-boundary soft-mode in the Cmcm phase is gradually frozen in as $\mathrm{T_C}$ is approached from above. This leads to d_2 increasing and d_3 decreasing, as well as a shift between the top layer and bottom layer in the Pnma structure (Supplementary Figure 27-a). The temperature dependence of phonon eigenvectors obtained from the linearly-interpolated $\Phi^{(2)}(T)$ described above is shown in Supplementary Figure 27-b. Below $\mathrm{T_C}$, the Sn c element is the dominant component of the TO_c eigenvector. As temperature rises, the overall displacement amplitude of Sn increases while that of S decreases. The increase in Sn amplitude is from the c component. Meanwhile, the amplitude of S atoms decreases due to its decreasing c component.

2. SUPPLEMENTARY TABLES

T_{BE}	$\Phi^{(2)}$	TO_c/A_g^0 (meV)	A_g^1 (meV)	B_{3g}^0 (meV)
300 K	DFT	0.21 $(0.15 \pm 0.03$ at 295 K)	0.38 $(0.33 \pm 0.03$ at 295 K)	0.12 $(0.16 \pm 0.03$ at 295 K)
600 K	DFT	0.43	0.74	0.24
846 K	DFT	0.60	1.0.5	0.34
846 K	TDEP 600 K	3.33 $(1.03 \pm 0.18$ at 853 K)	2.06 $(1.84 \pm 0.016$ at 844 K)	0.38 $(0.43 \pm 0.20$ at 696 K)

Supplementary Table 1: A comparison of linewidths from simulations (no parentheses) with those extracted from fitting Raman spectra (in parentheses) at different temperatures. Simulated linewidths have different T_{BE} and $\Phi^{(2)}$ but all were calculated from almaBTE using $\Phi^{(3)}$ from DFT.

Bonds	0 K Pnma	600 K Pnma	800 K Cmcm
d_1	$\begin{pmatrix} -3.34 & - & 0.02 \\ - & -0.46 & - \\ -0.21 & - & -0.30 \end{pmatrix}$	$\begin{pmatrix} -2.54 & - & 0.09 \\ - & -0.32 & - \\ 0.13 & - & -0.18 \end{pmatrix}$	$\begin{pmatrix} -2.55 & - & - \\ - & -0.21 & - \\ - & - & -0.21 \end{pmatrix}$
d_2	$\begin{pmatrix} -0.49 & -0.04 & -0.17 \\ -0.22 & -1.25 & -0.94 \\ -0.35 & -1.03 & -1.70 \end{pmatrix}$	$\begin{pmatrix} -0.35 & -0.02 & -0.08 \\ -0.15 & -0.87 & -0.64 \\ -0.23 & -0.71 & -1.10 \end{pmatrix}$	$\begin{pmatrix} -0.20 & - & -0.06 \\ -0.16 & -0.32 & -0.17 \\ -0.16 & -0.21 & -0.34 \end{pmatrix}$
d_3	$\begin{pmatrix} -0.07 & 0.02 & -0.02 \\ -0.08 & -0.03 & -0.21 \\ 0.01 & -0.19 & 0.18 \end{pmatrix}$	$\begin{pmatrix} -0.03 & -0.03 & -0.06 \\ -0.04 & -0.06 & -0.14 \\ 0.04 & -0.12 & 0.11 \end{pmatrix}$	$\begin{pmatrix} -0.20 & - & 0.06 \\ -0.16 & -0.32 & 0.16 \\ 0.16 & 0.21 & -0.34 \end{pmatrix}$

Supplementary Table 2: Second order force constants (eV/Å^2) for the bonds important in the Pnma-Cmcm distortion from 0 K (DFT) and 600 K (AIMD) in the Pnma phase and 800 K (AIMD) in the Cmcm phase.

Pnma	a	b	c	Cmcm	a	b	c	Pnma 600 K	a	b	c	Cmcm 800 K	a	b	c
	10.97 Å	3.95 Å	4.19 Å		11.16 Å	4.00 Å	4.00 Å		10.97 Å	3.95 Å	4.19 Å		11.16 Å	4.00 Å	4.00 Å
	X	Y	Z		X	Y	Z		X	Y	Z		X	Y	Z
Sn	0.118	0.250	0.109	Sn	0.122	0.250	0.000	Sn	0.121	0.247	0.098	Sn	0.118	0.238	-0.004
Sn	0.382	0.750	0.609	Sn	0.378	0.750	0.500	Sn	0.384	0.752	0.591	Sn	0.376	0.738	0.512
Sn	0.618	0.250	0.391	Sn	0.622	0.250	0.500	Sn	0.619	0.241	0.415	Sn	0.623	0.247	0.495
Sn	0.882	0.750	0.891	Sn	0.878	0.750	0.000	Sn	0.885	0.761	0.894	Sn	0.878	0.789	-0.006
S	0.146	0.750	0.526	S	0.147	0.750	0.500	S	0.146	0.747	0.525	S	0.142	0.738	0.491
S	0.354	0.250	0.026	S	0.353	0.250	0.000	S	0.358	0.244	0.030	S	0.351	0.239	-0.003
S	0.646	0.750	0.974	S	0.647	0.750	0.000	S	0.647	0.741	0.975	S	0.646	0.763	0.005
S	0.854	0.250	0.474	S	0.852	0.250	0.500	S	0.856	0.257	0.471	S	0.854	0.254	0.508

Supplementary Table 3: Atomic fractional internal coordinates for different calculations of the SnS crystal structure. From left to right: Pnma phase DFT, Cmcm phase DFT, 600 K AIMD in the Pnma phase and 800 K AIMD in the Cmcm phase.

3. SUPPLEMENTARY FIGURES

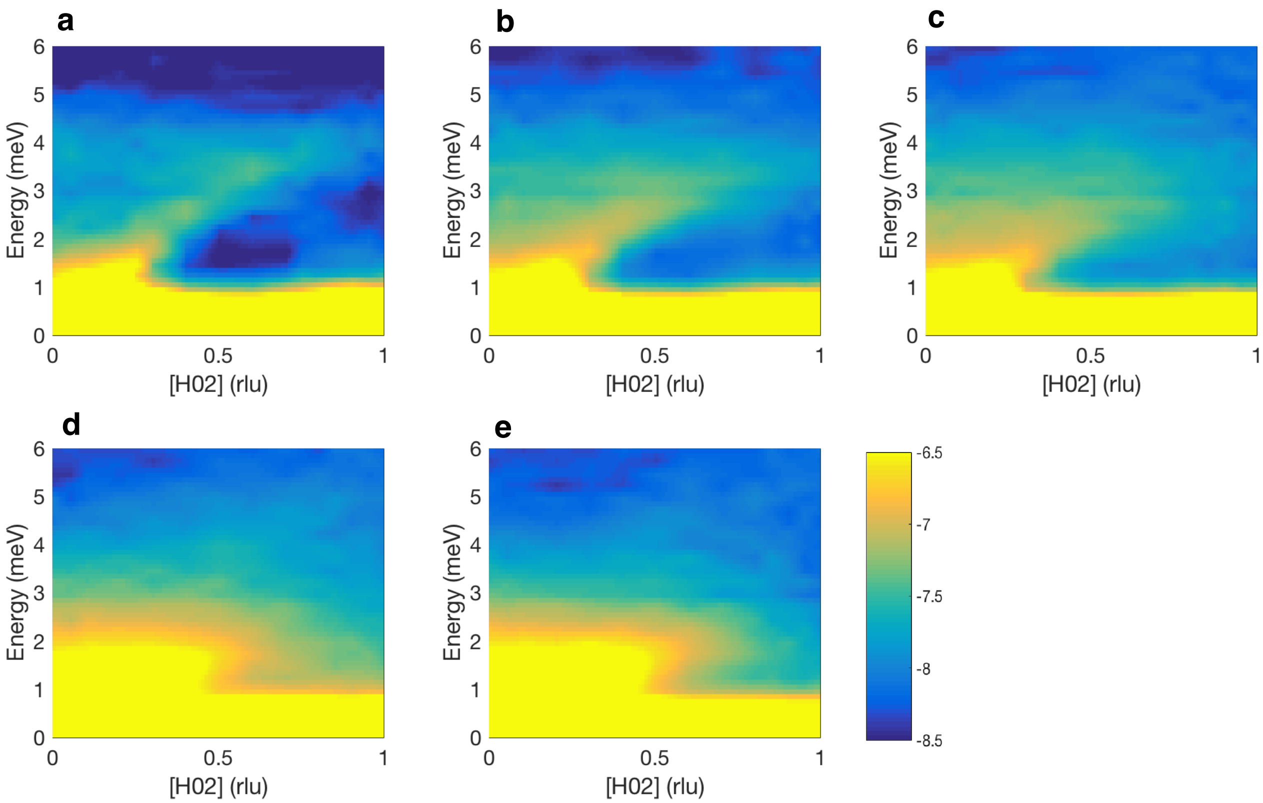

Supplementary Figure 1: Temperature evolution of transverse phonon dispersions ($S(\mathbf{Q}, E)$) along [100] across the structural phase transition as measured with INS on SnSe. Temperatures shown are 500 K (a), 700 K (b), 770 K (c), 850 K (d) and 990 K (e). These reciprocal-space paths highlight c polarized low-energy TA and TO phonons in the Pnma phase (a-c) and TA phonons in the Cmcm phase (d-e). Data were collected on HB-3 at HFIR. Intensity is plotted in a $\log_{10}$ scale.

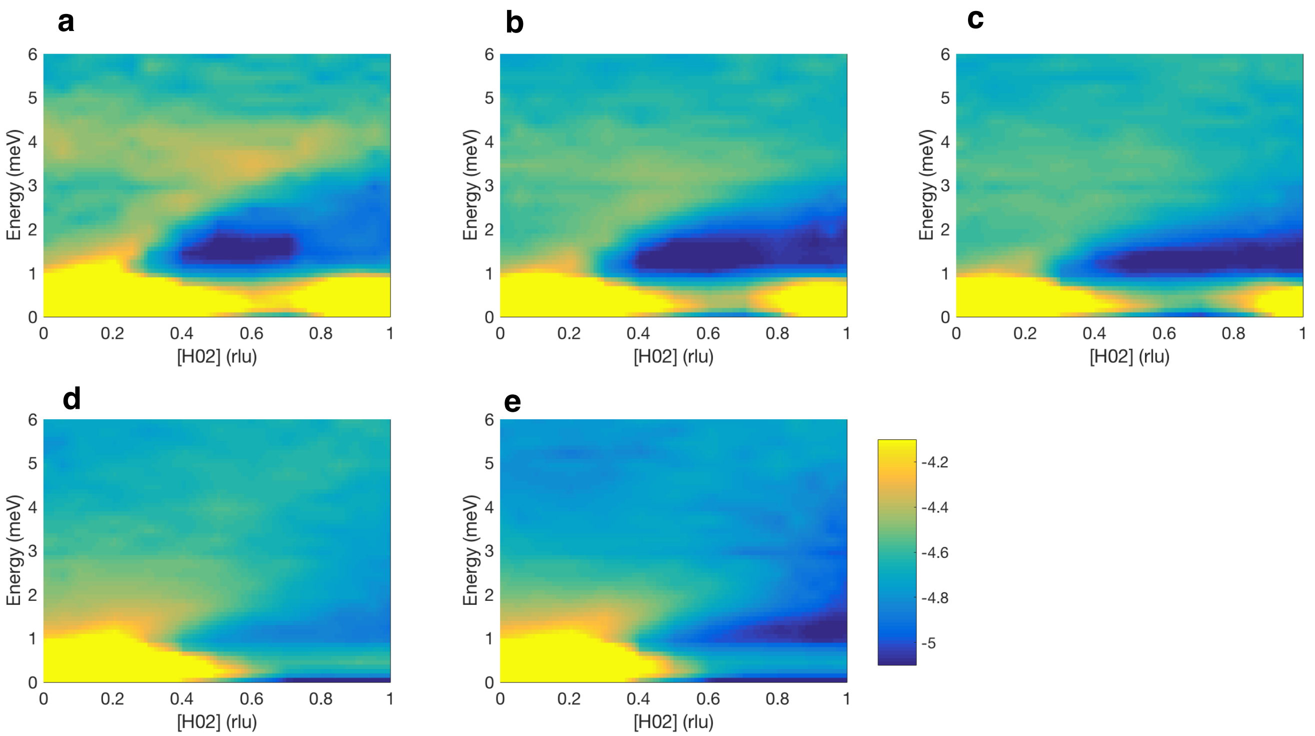

Supplementary Figure 2: Temperature evolution of transverse phonon dispersions ($\chi''(\mathbf{Q}, E)$) along [100] across the structural phase transition as measured with INS on SnSe. Temperatures shown are 500 K (a), 700 K (b), 770 K (c), 850 K (d) and 990 K (e). These reciprocal-space paths highlight c polarized low-energy TA and TO phonons in the Pnma phase (a-c) and TA phonons in the Cmcm phase (d-e). Data were collected on HB-3 at HFIR. Intensity is plotted in a $\log_{10}$ scale.

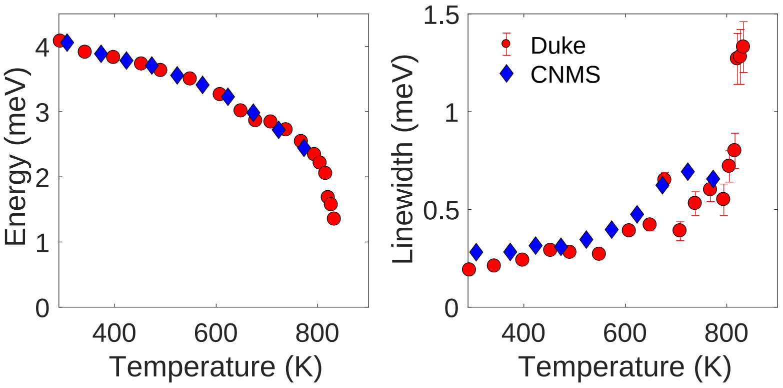

Supplementary Figure 3: Phonon energy (left) and linewidth (right) extracted from SnSe Raman data for the soft-mode (A_g^0). Good agreement is found between data measured at Duke University and at the Center for Nanophase Materials Science (CNMS) at ORNL. Errorbars represent a 95% confidence interval from the fitting procedure.

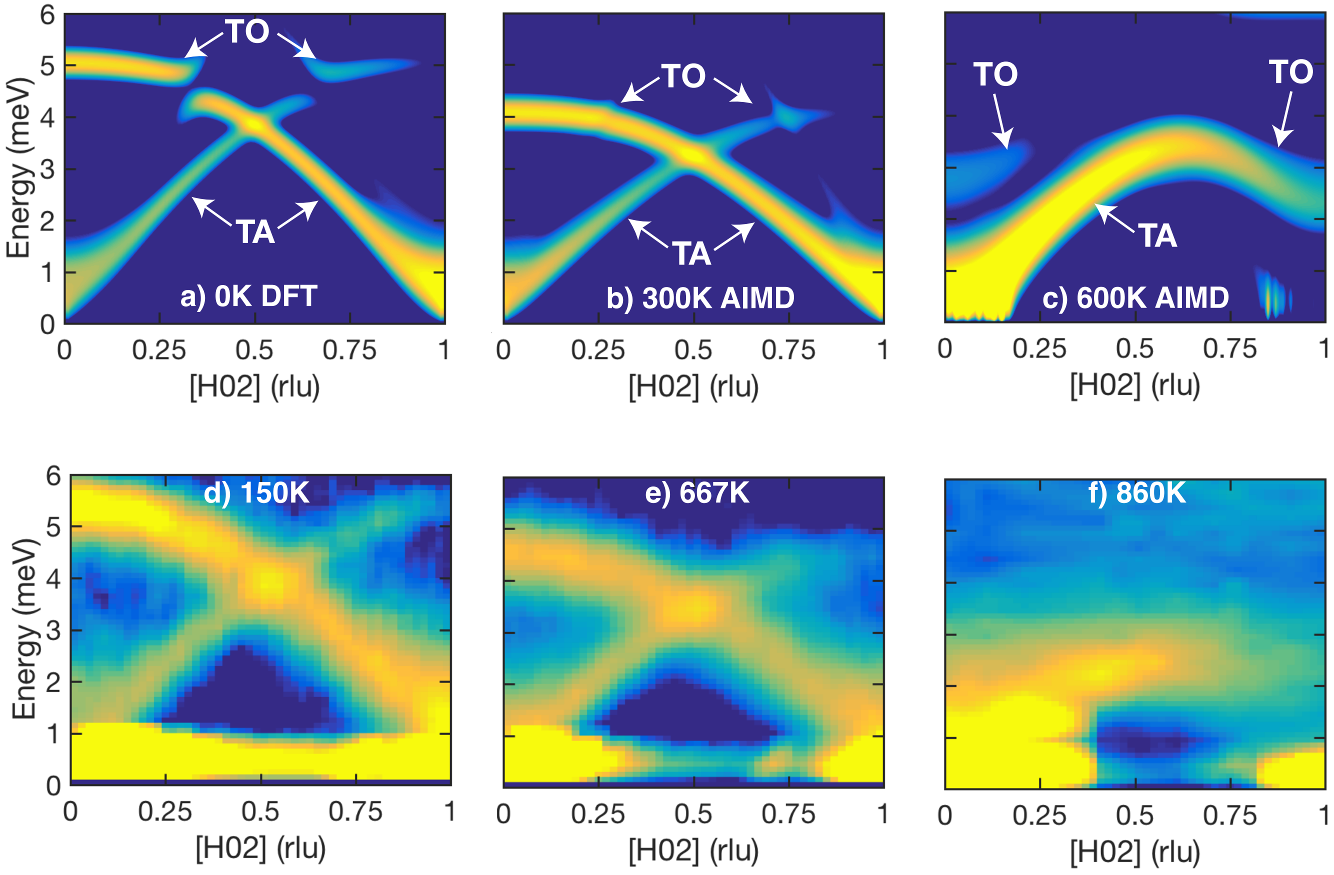

Supplementary Figure 4: Temperature evolution of transverse phonon dispersions in SnS along [100] across the structural phase transition. Simulated $\chi''(\mathbf{Q}, E)$ from harmonic second-order force constants (a) and renormalized second-order force constants from AIMD (b-c) show the c-polarized TA and TO modes as indicated. Panels (d-e) show experimental $\chi''(\mathbf{Q}, E)$ measured on CNCS whilst (f) is from HB-3. Intensity is plotted in a $\log_{10}$ scale.

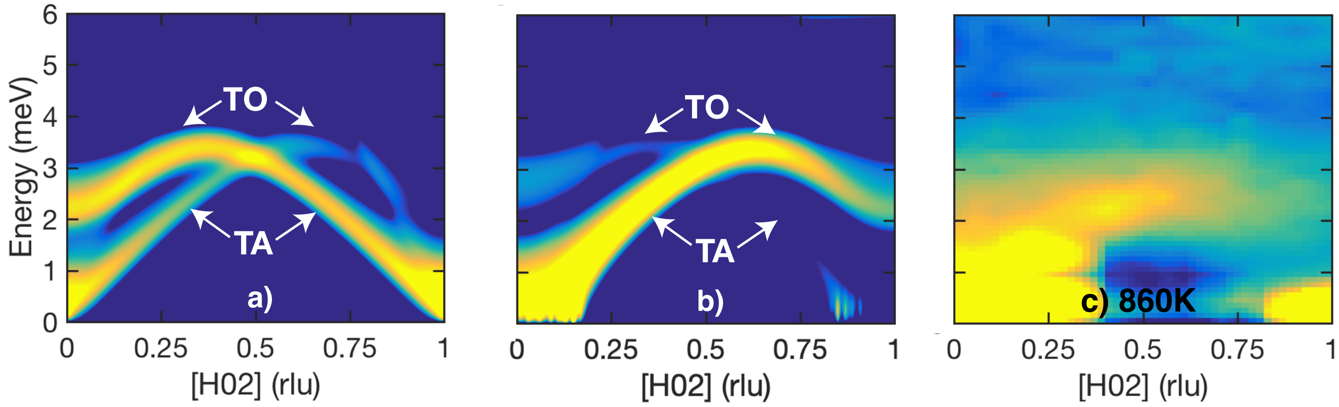

Supplementary Figure 5: As SnS changes from Pnma to Cmcm there is a continuous evolution of internal coordinates (r_d) which impacts the $\chi''(\mathbf{Q}, E)$ intensities. Here we show $\chi''(\mathbf{Q}, E)$ calculated using renormalized second-order force constants from 600 K AIMD (a-b) with internal coordinates from 0 K DFT for the Pnma phase (a) and for the Cmcm phase (b). Panels (c) shows experimental data from HB-3. Intensity is plotted in a $\log_{10}$ scale.

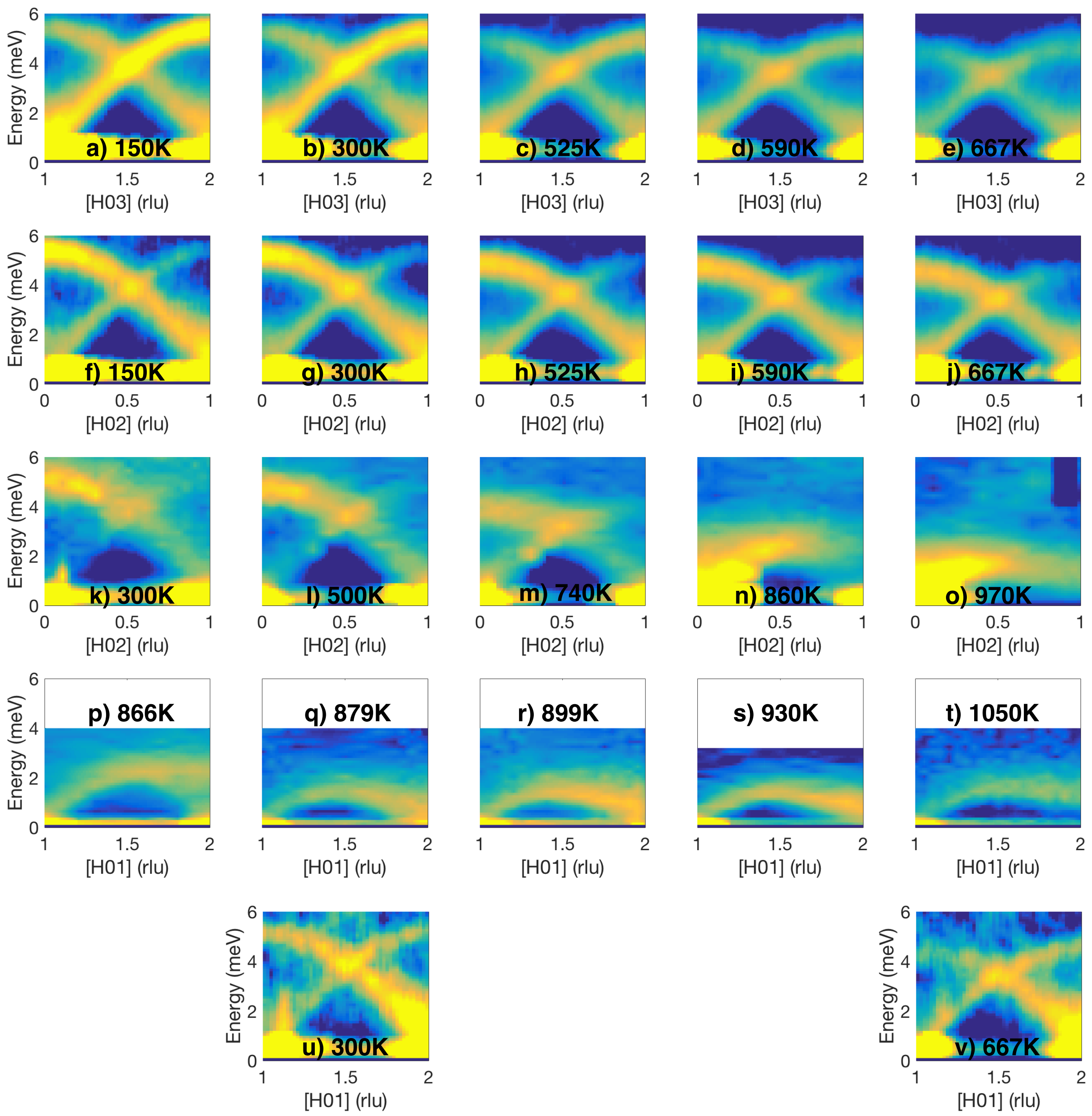

Supplementary Figure 6: Temperature evolution of transverse phonon dispersions in SnS along [100] across the structural phase transition as measured with INS. Panels a-e are plotted along [H03], panels f-o along [H02] and the [H01] direction is shown in p-v. These reciprocal-space paths highlight low-energy TA and TO phonons polarized along c in the Pnma phase (a-n and u-v) and Cmcm phase (o-t). Data were collected on CNCS (a-j, and u-v), HB-3 (k-o) and CTAX (p-t). Intensity is plotted in a $\log_{10}$ scale.

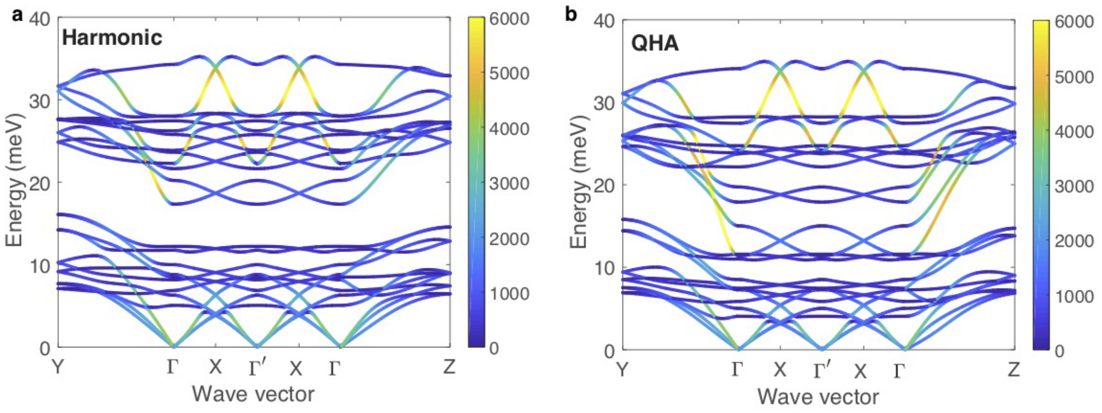

Supplementary Figure 7: SnS phonon dispersions for the Pnma structure. (a) $\Phi^{(2)}$ from the harmonic approximation and (b) $\Phi^{(2)}$ from QHA, with lattice parameters corresponding to 862 K (details in methods). Color scale indicates the group velocities (m/s).

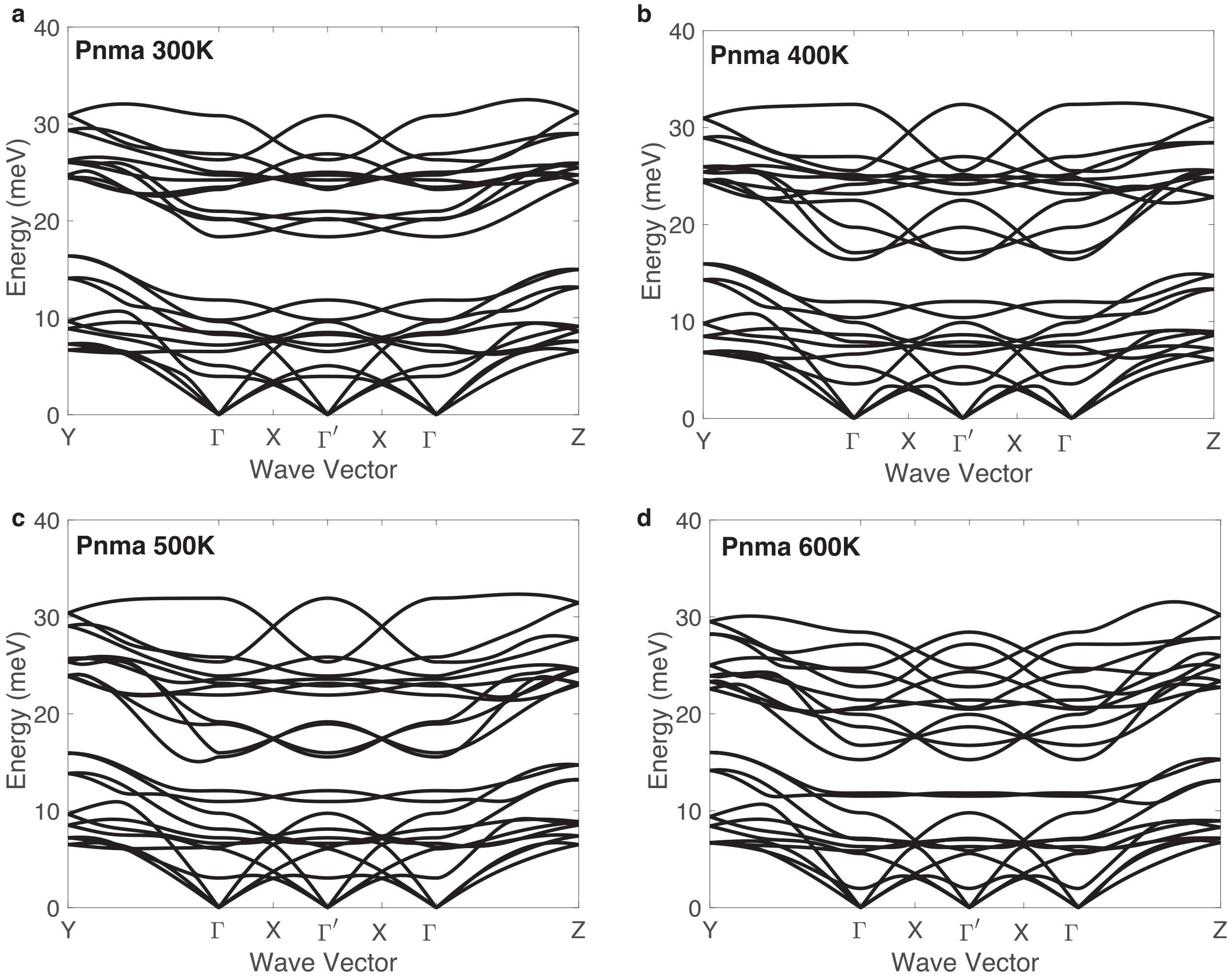

Supplementary Figure 8: SnS phonon dispersions for the Pnma structure. AIMD was performed at 300, 400, 500 and 600 K to extract renormalized $\Phi^{(2)}$ using an interaction cutoff distance of $r = 7.887$ Å within the TDEP method. The reciprocal path plotted here is equivalent to that in Supplementary Figure 9. $\Gamma^{'}$ is a superlattice zone center in the Pnma phase and Γ-X-$\Gamma^{'}$ in Pnma corresponds to Γ-Y direction in the Cmcm phase.

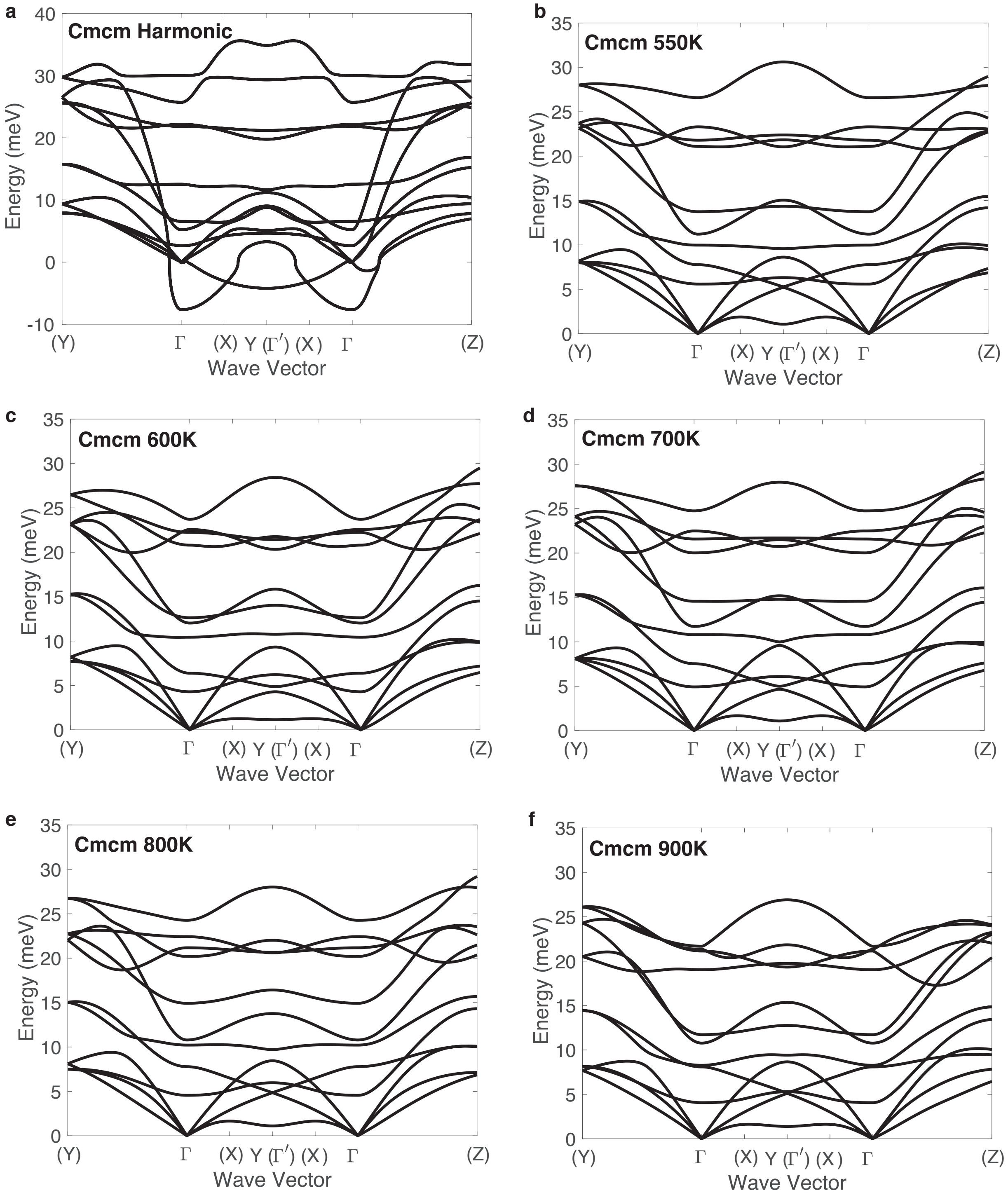

Supplementary Figure 9: SnS phonon dispersions for the Cmcm structure. (a) $\Phi^{(2)}$ from the harmonic approximation (b-f) 550, 600, 700, 800 and 900 K AIMD with renormalized $\Phi^{(2)}$ extracted with using interaction cutoff distance of $r = 6.954\,\text{Å}$ within the TDEP method. The reciprocal path plotted here is equivalent to that in Supplementary Figure 8. $\Gamma^{'}$ is a superlattice zone center in the Pnma phase and Γ-X-$\Gamma^{'}$ in Pnma corresponds to Γ-Y direction in the Cmcm phase.

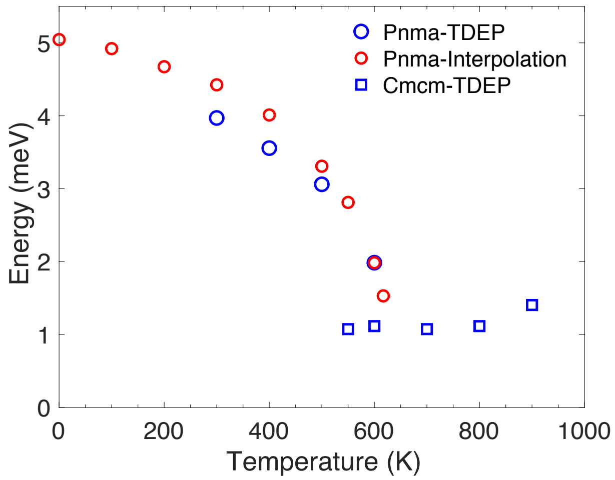

Supplementary Figure 10: SnS phonon dispersions were calculated for the Pnma phase at 300, 400, 500 and 600 K and extracted with an interaction cutoff distance of $r = 7.887$ Å, and for the Cmcm phase at 550, 600, 700, 800 and 900 K extracted with an interaction cutoff distance of $r = 6.954$ Å in the TDEP method. TO_c and TA_c mode frequencies at Γ were extracted from dispersions in the Pnma and Cmcm phases respectively. In addition, a linear interpolation between the harmonic and renormalized $\Phi^{(2)}$ at 600 K was used to approximate the temperature dependence of TO_c mode frequencies and extrapolated to the phase transition. The highest temperature to which the Pnma phase retains stable phonon dispersions is 617 K using this linear interpolation method.

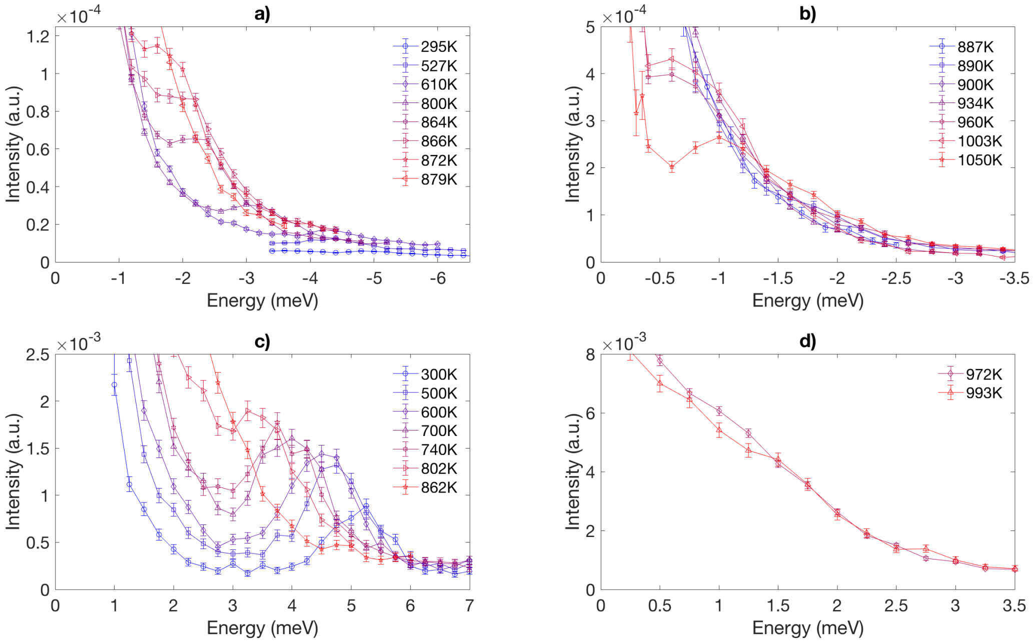

Supplementary Figure 11: S(E) spectra showing soft mode behavior in SnS as measured on CTAX for $\mathbf{Q} = (2,0,1)$ (panels a and b) and HB-3 at $\mathbf{Q} = (0,0,2)$ (panels c and d). CTAX data was measured on the neutron energy gain side whereas HB-3 data was collected on the neutron energy loss side. The Pnma phase is shown in a and c whilst the Cmcm phase is seen in b and d. Error bars are from Poisson statistics and represent one standard deviation.

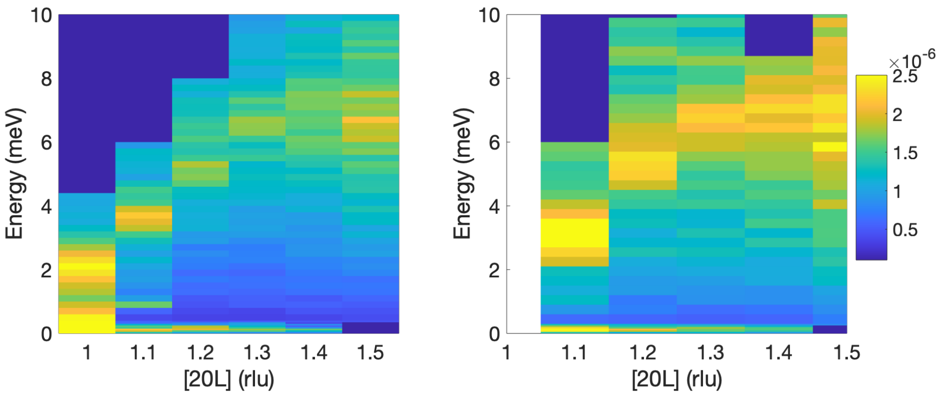

Supplementary Figure 12: $\chi''(\mathbf{Q}, E)$ showing soft mode behavior in SnS along [001] as measured on CTAX along $[2, 0, L]$ at 866 K (left) and 899 K (right). Intensity is plotted in a linear scale.

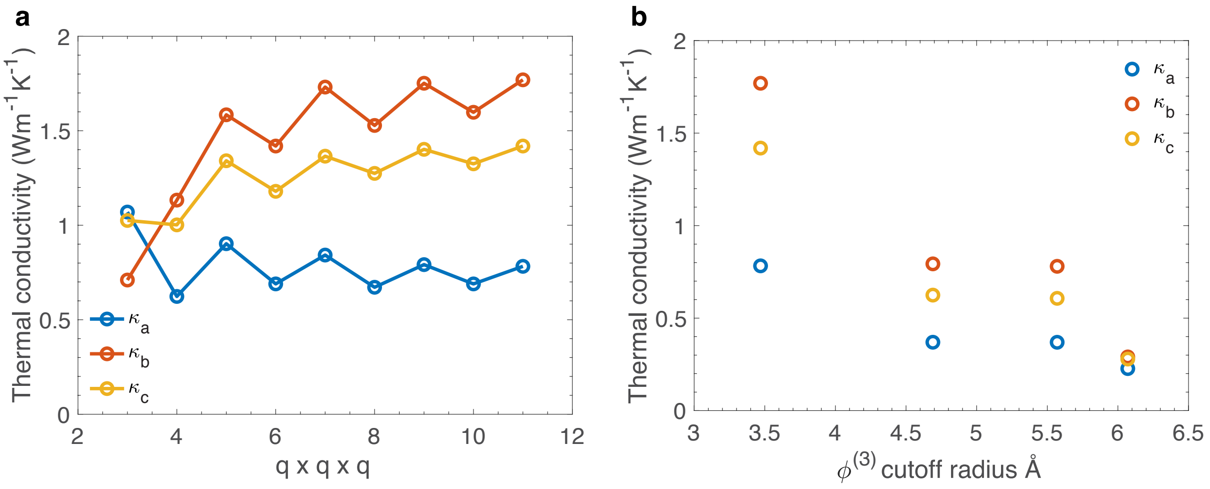

Supplementary Figure 13: Convergence test of **q**-point mesh and $\Phi^{(3)}$ cutoff radius. The convergence for third order force constants was studied using a 2x4x4 supercell, 2×3×3 Γ centered **k**-point, 500 eV cutoff energy and total energy for each configuration was converged to 10^{-4} eV. (a) Thermal conductivity convergence of **q**-point mesh with $\Phi^{(3)}$ cutoff radius being 3.47 Å and harmonic $\Phi^{(2)}$ at $T_{BE} = 300$ K. (b) Thermal conductivity convergence of cutoff radius using an 11×11×11 **q**-point mesh and harmonic $\Phi^{(2)}$ at $T_{BE} = 300$ K. With 11×11×11 **q**-point mesh, thermal conductivities show convergence already, and total thermal conductivities behave similar as SnSe in [9] with increasing cutoff radius. For the final production calculations, a more converged set of DFT settings was used, with total energy converged to 10^{-8} eV (details in Methods of main text).

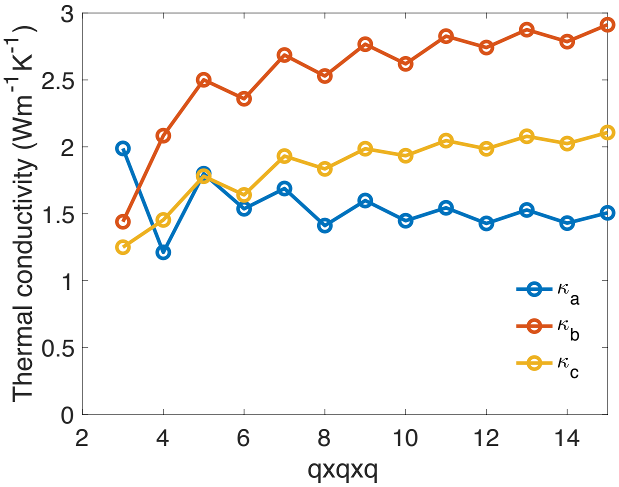

Supplementary Figure 14: Convergence test of **q**-point mesh for TDEP thermal conductivity calculations. Thermal conductivity calculations were done with TDEP and using 300K AIMD $\Phi^{(2)}$ and $\Phi^{(3)}$ extracted by TDEP. The thermal conductivity calculations were finished with the adaptive Gaussian integration method at $T_{BE} = 300\,K$.

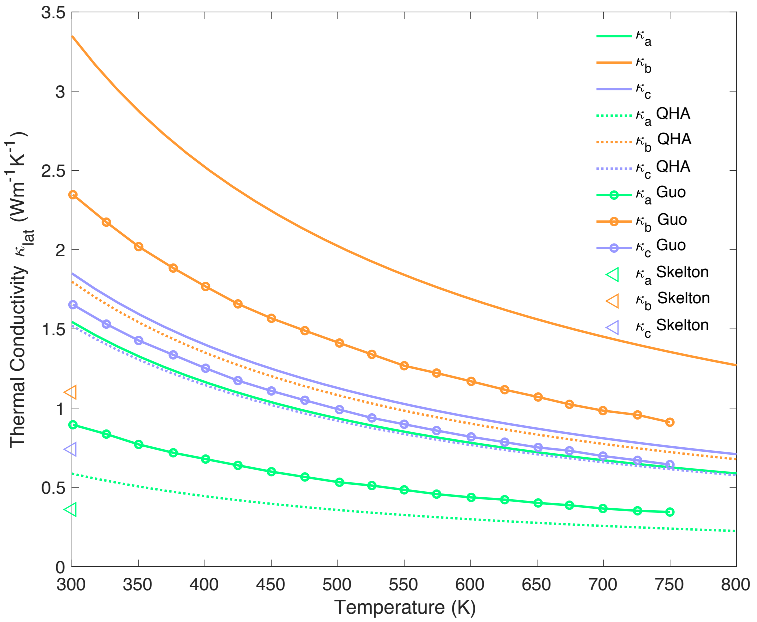

Supplementary Figure 15: Simulated SnS κ_{lat} along different directions are calculated using $0\,K$ $\Phi^{(2)}$ from both the harmonic and quasi-harmonic approximations (QHA). Calculated κ_{lat} are from Guo *et al.* [10] and Skelton *et al.* [11] are shown here as well.

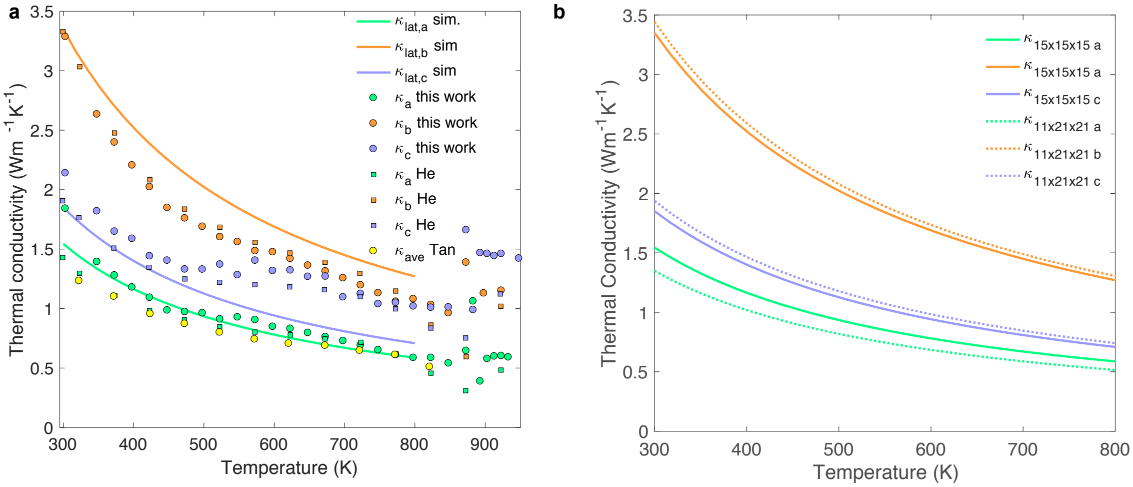

Supplementary Figure 16: Experimental SnS thermal conductivities along the a, b and c crystallographic directions. (a) Literature single crystal data is from He *et al.* [12] and polycrystalline data is from Tan *et al.* [13]. Simulated κ_{lat} along different directions are calculated using $0\,\mathrm{K}\ \Phi^{(2)}$ from the harmonic approximation, and the phonon **q**-point mesh for thermal conductivity calculation was 15×15×15. (b) A comparison of using a phonon **q**-point mesh for thermal conductivity calculation of 15×15×15 versus 11×21×21 is shown.

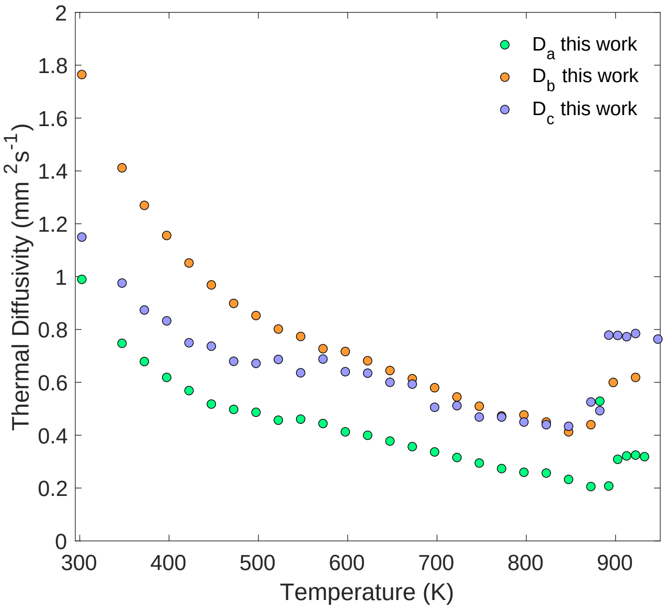

Supplementary Figure 17: Thermal diffusivity measured along the a, b and c crystallographic directions of SnS using the light flash method.

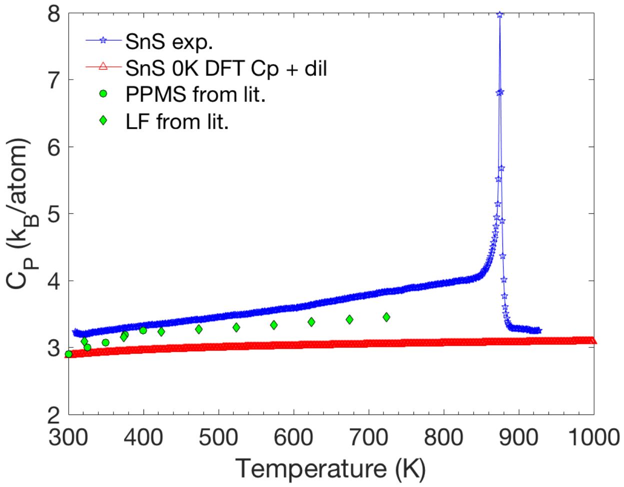

Supplementary Figure 18: Heat capacity of SnS from DSC and 0 K DFT (includes correction for sample dilation with temperature) compared to experimental results by Tan *et al.* which used a physical property measurement system (PPMS) and the light flash (LF) technique [13].

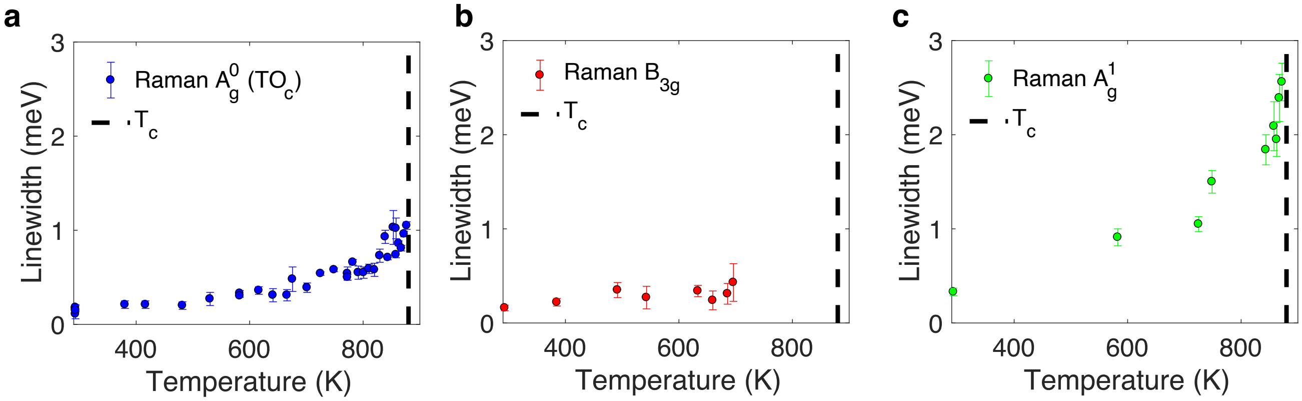

Supplementary Figure 19: Phonon linewidths extracted from fitting Raman spectra of different modes in SnS. Error bars represent a 95% confidence interval from the fitting procedure.

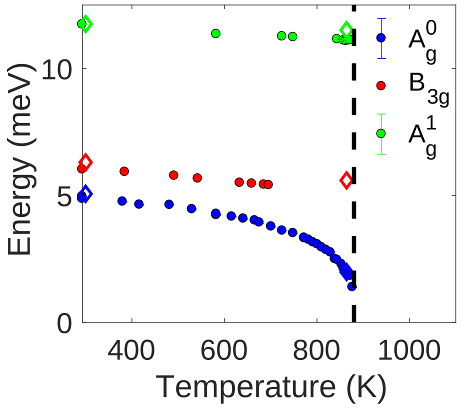

Supplementary Figure 20: Phonon energies extracted from fitting Raman spectra of different modes in SnS (circles). Diamond markers are phonon energies from DFT simulations (see main text). Error bars represent a 95% confidence interval from the fitting procedure.

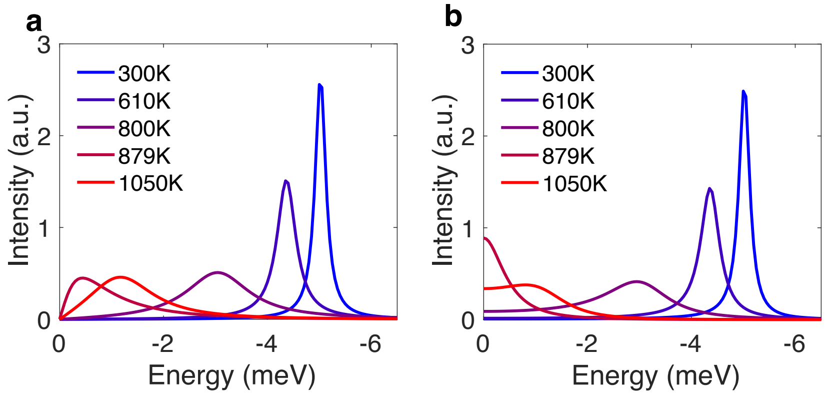

Supplementary Figure 21: DHO profiles extracted from fits to SnS CTAX experimental data for the TO_c mode at **Q** = (2,0,1): (a) $\chi''(E)$ and (b) $S(E)$. All DHO profiles are normalized to a unit integral.

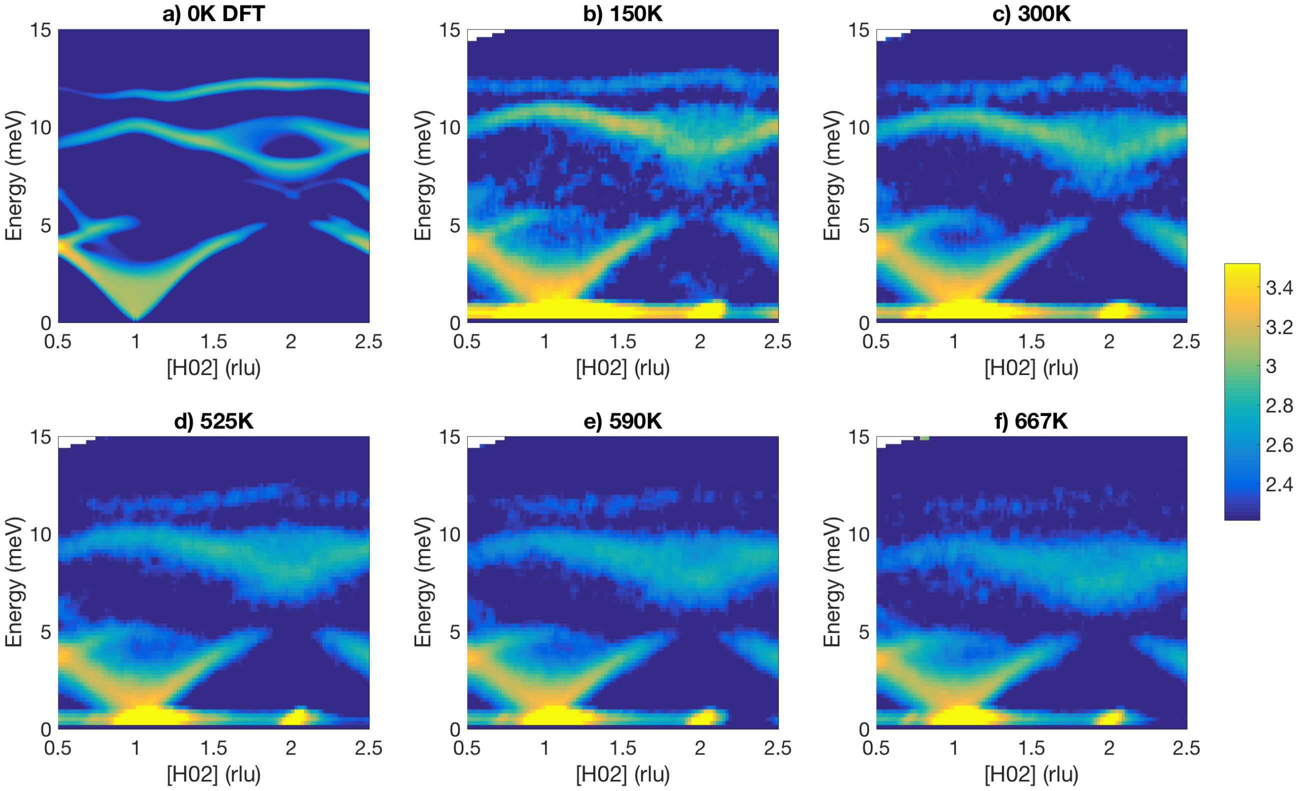

Supplementary Figure 22: $\chi''(\mathbf{Q}, E)$ slices for SnS along $[H02]$ in the Pnma phase, measured with INS (CNCS) at the indicated temperatures and calculated with DFT. Intensity is plotted in a $\log_{10}$ scale.

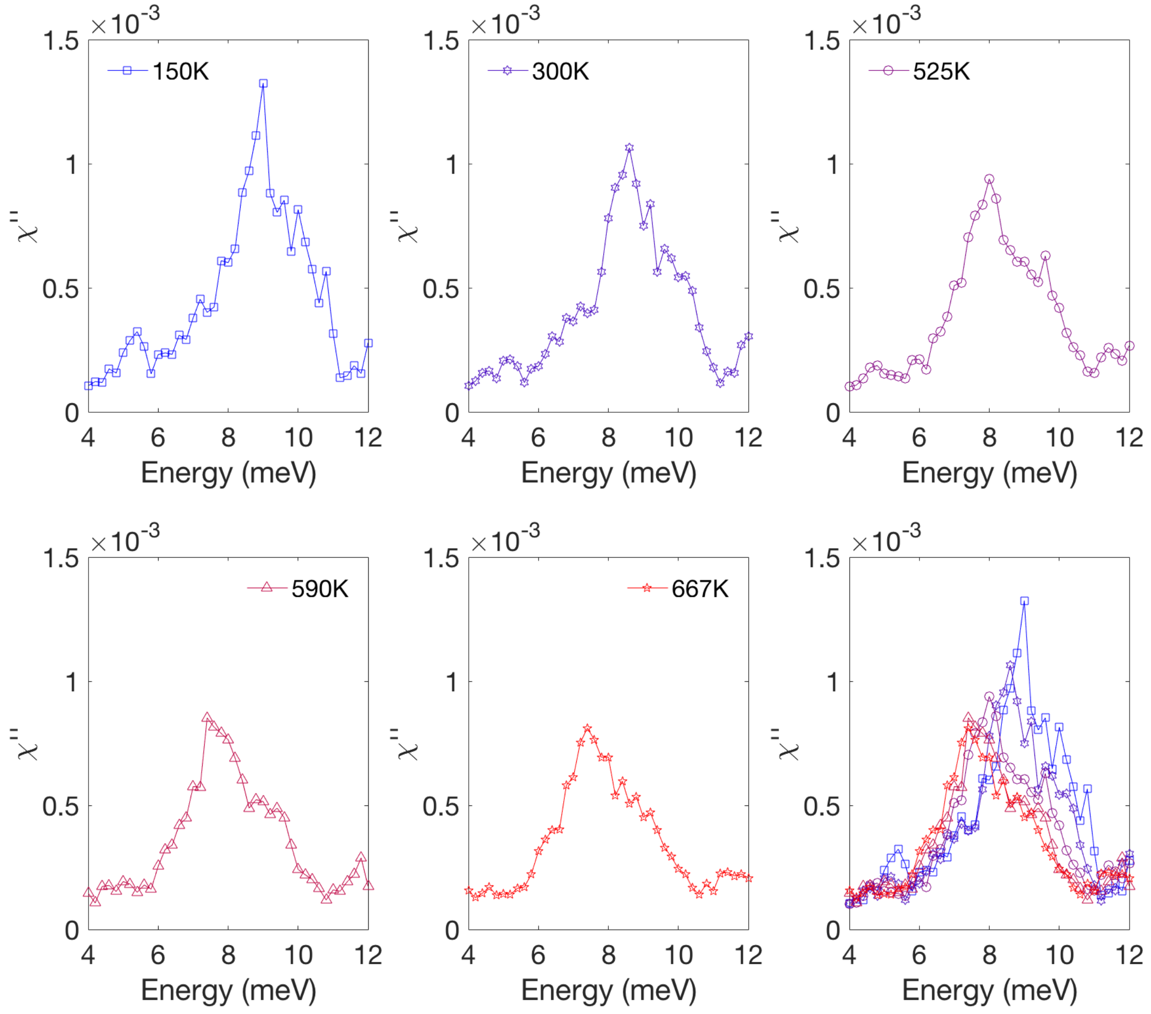

Supplementary Figure 23: Temperature evolution of $\chi''(E)$ for optical phonons measured at $\mathbf{Q}$ = (2,0,-2) in SnS on CNCS.

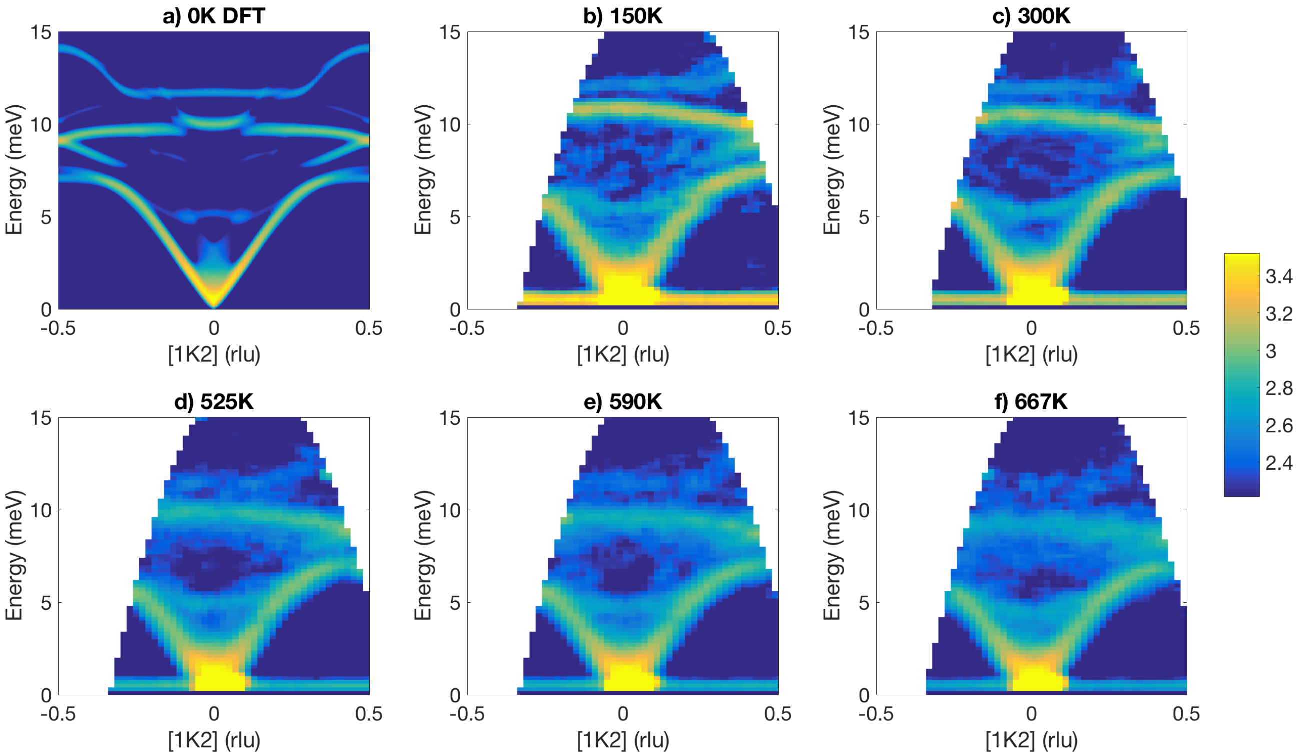

Supplementary Figure 24: $\chi''(\mathbf{Q}, E)$ slices for SnS along $[1K2]$ in the Pnma phase, measured with INS (CNCS) at the indicated temperatures and calculated with DFT. Intensity is plotted in a $\log_{10}$ scale.

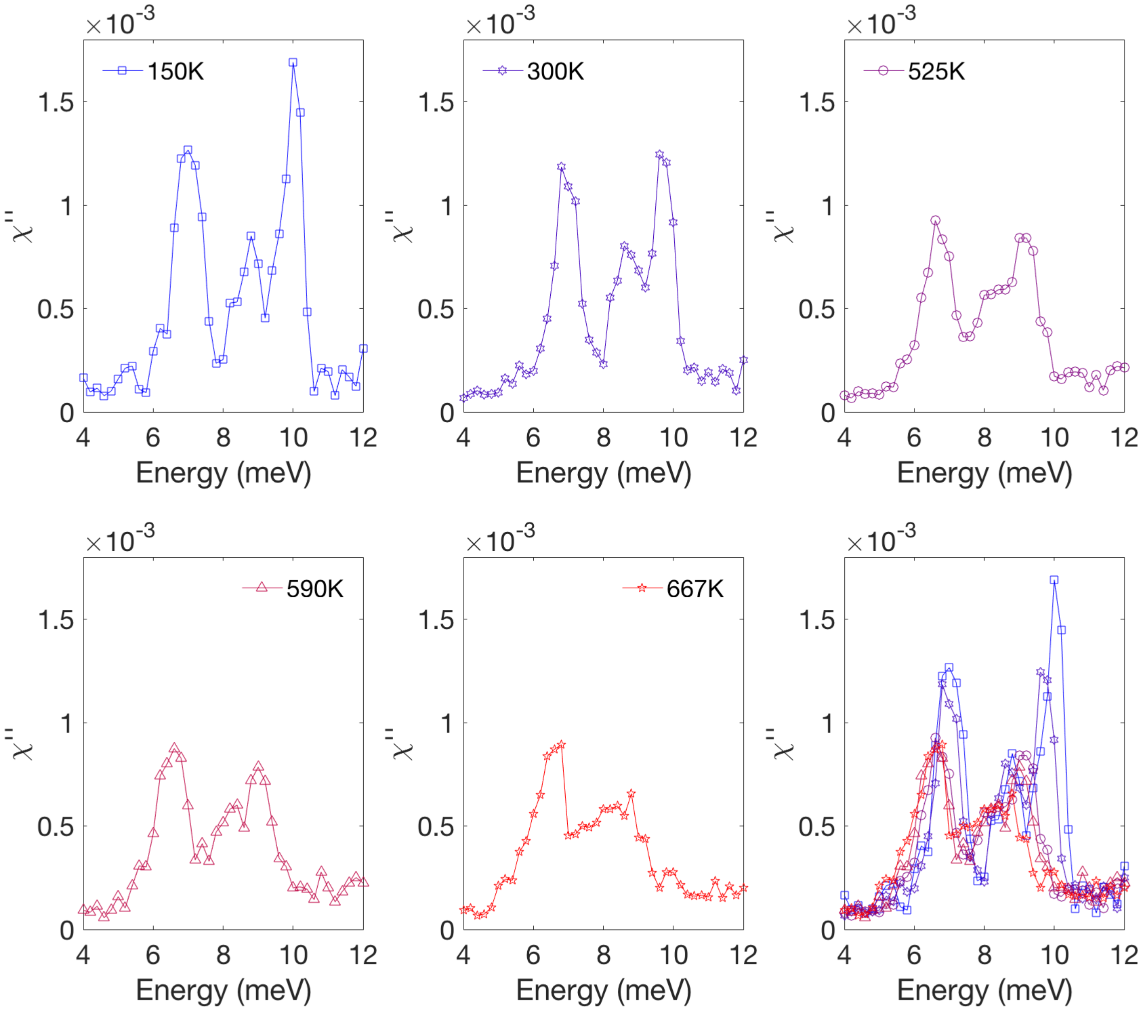

Supplementary Figure 25: Temperature evolution of $\chi''(E)$ for phonons measured at $\mathbf{Q}$ = (1,0.35,-2) in SnS on CNCS.

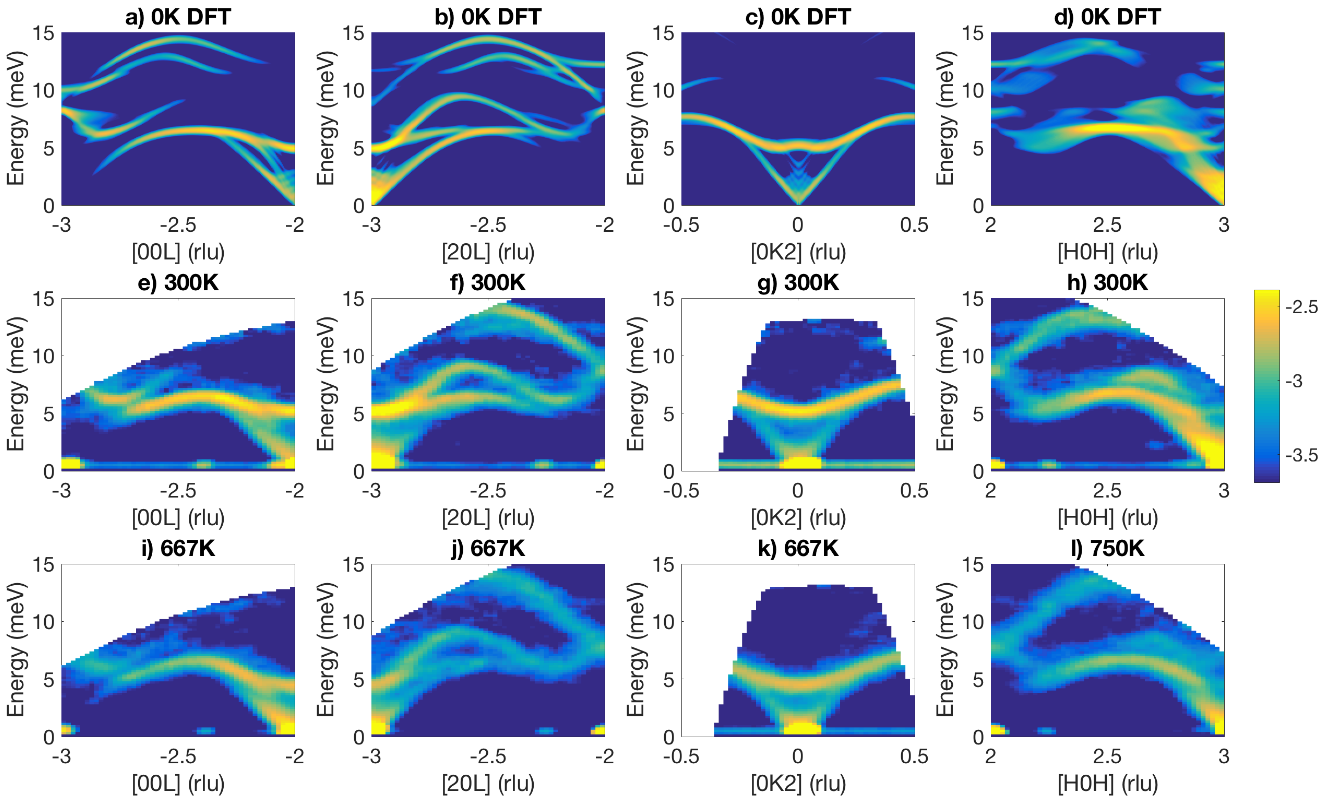

Supplementary Figure 26: $\chi''(\mathbf{Q}, E)$ slices for SnS in the Pnma phase, along different paths in reciprocal space, showing a strong anisotropy in the phonon dispersions. (a-d) First-principles simulations in harmonic approximation. (e-h) INS data from CNCS at 300 K. (i-l) INS data from CNCS at 750 K. Intensity is plotted in a $\log_{10}$ scale.

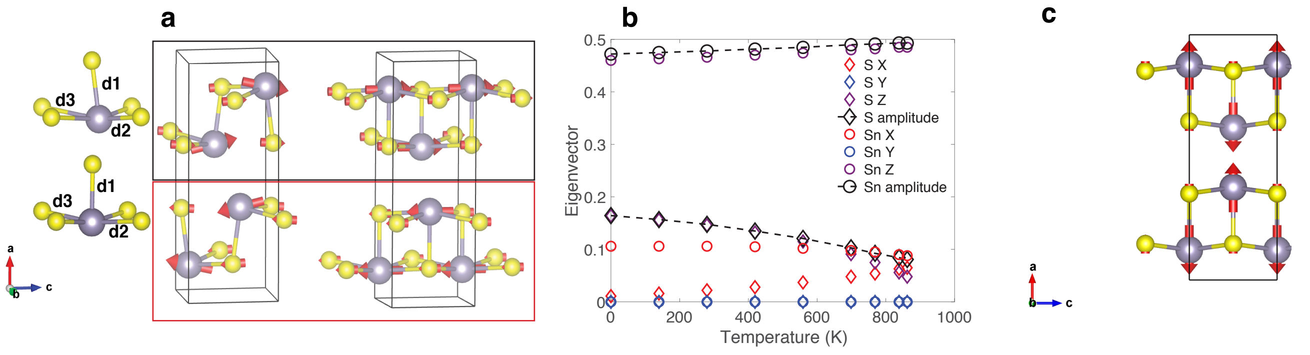

Supplementary Figure 27: (a) Eigenvectors of the soft phonon mode in the Pnma (left) and Cmcm (right) phases of SnS. The top layer is highlighted by the black frame and the bottom layer is highlighted by the red frame. Amplitudes of the TO_c eigenvector components are shown in (b). Temperatures have been scaled as described in the main text. (c) Eigenvector of the A_g mode in Cmcm phase as in SnSe [8].

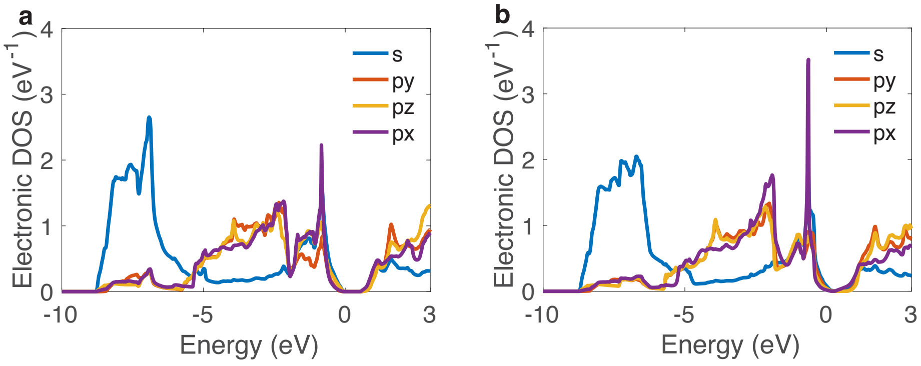

Supplementary Figure 28: Electronic partial density of states (DOS) for SnS calculated with DFT for the relaxed (a) Pnma and (b) Cmcm structures.

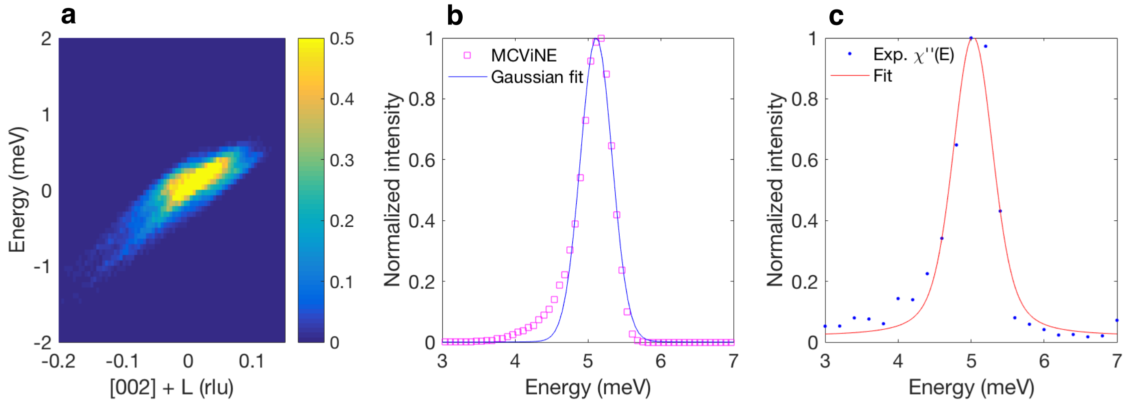

Supplementary Figure 29: (a) Resolution function R($\mathbf{Q}$,E) for CNCS along $[0, 0, 2 + L]$ as calculated by virtual neutron simulations with MCViNE using the same instrument parameters as CNCS measurements. (b) $R(E)$ summed over all $\mathbf{Q}$ as evaluated from MCViNE was fit to a Gaussian. (c) Convolution of damped harmonic oscillator and gaussian fit to experimental $\chi''(E)$. Gaussian parameters are taken from fits to MCViNE simulation whilst damped harmonic oscillator components are fitted to the data.

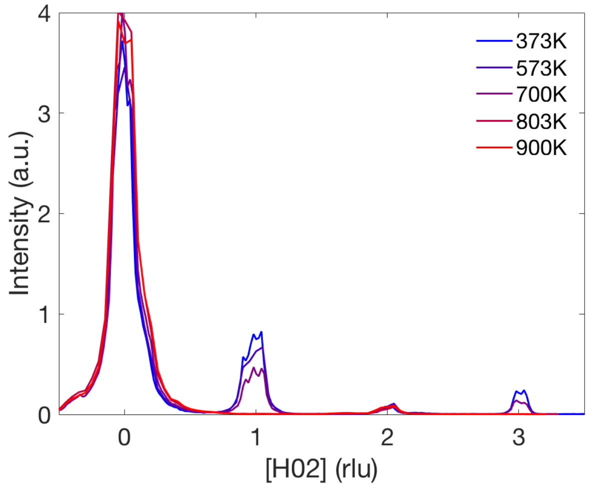

Supplementary Figure 30: Temperature evolution of diffraction along $[H02]$ in SnSe as measured on HB-3. Superstructure peak intensities at (102) and (302) are fully suppressed in the Cmcm phase.

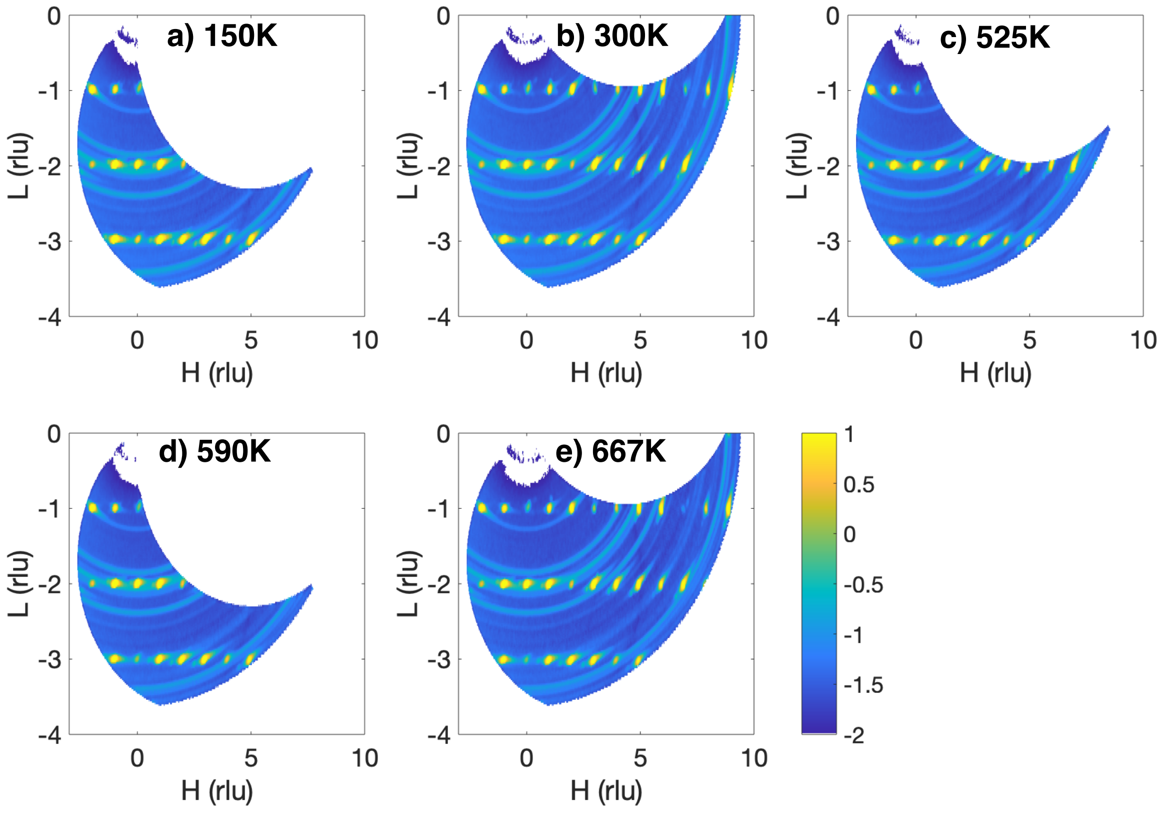

Supplementary Figure 31: Temperature evolution of elastic scattering ($S(\mathbf{Q}, E = 0)$) in the $(H0L)$ plane. Energy was integrated $\pm$ 1 meV and was measured on SnS using the CNCS. Intensity is plotted in a $\log_{10}$ scale.

[1] Adouby, K., Perez-Vicente, C. & Jumas, J. Structure and temperature transformation of SnSe. Stabilization of a new cubic phase $Sn_4Bi_2Se_7$. *Z. Kristallogr* **213**, 343–349 (1998).

[2] Chattopadhyay, T., Werner, A., Von-Schnering, H. & Pannetier, J. Temperature and pressure induced phase transition in IV-VI compounds. *Revue de Physique Appliquee* **19**, 807–813 (1984).

[3] Wiedemeier, H. & Csillag, F. J. The thermal expansion and high temperature transformation of SnS and SnSe. *Zeitschrift für Kristallographie-Crystalline Materials* **149**, 17–30 (1979).

[4] Tremel, W. & Hoffmann, R. Tin sulfide, $(Te_2)_2I_2$, and related compounds: Symmetry-controlled deformations in solid-state materials. *Inorganic Chemistry* **26**, 118–127 (1987).

[5] Kroumova, E. *et al.* Bilbao crystallographic server: useful databases and tools for phase-transition studies. *Phase Transitions: A Multinational Journal* **76**, 155–170 (2003).

[6] Thapa, J., Liu, B., Woodruff, S. D., Chorpening, B. T. & Buric, M. P. Raman scattering in single-crystal sapphire at elevated temperatures. *Applied optics* **56**, 8598–8606 (2017).

[7] Bansal, D. *et al.* Phonon anharmonicity and negative thermal expansion in SnSe. *Physical Review B* **94**, 054307 (2016).

[8] Hong, J. & Delaire, O. Phase transition and anharmonicity in SnSe. *Materials Today Physics* **10**, 100093 (2019).

[9] Li, C. W. *et al.* Orbitally driven giant phonon anharmonicity in SnSe. *Nature Physics* **11**, 1063 (2015).

[10] Guo, R., Wang, X., Kuang, Y. & Huang, B. First-principles study of anisotropic thermoelectric transport properties of IV-VI semiconductor compounds SnSe and SnS. *Physical Review B* **92**, 115202 (2015).

[11] Skelton, J. M. *et al.* Lattice dynamics of the tin sulphides SnS_2, SnS and Sn_2S_3: vibrational spectra and thermal transport. *Physical Chemistry Chemical Physics* **19**, 12452–12465 (2017).

[12] He, W. *et al.* Remarkable electron and phonon band structures lead to a high thermoelectric performance ZT > 1 in earth-abundant and eco-friendly SnS crystals. *Journal of Materials Chemistry A* **6**, 10048–10056 (2018).

[13] Tan, Q. *et al.* Thermoelectrics with earth abundant elements: low thermal conductivity and high thermopower in doped SnS. *Journal of Materials Chemistry A* **2**, 17302–17306 (2014).
